# Evaluation of an integrated variable flip angle protocol to estimate coil B_1_ for hyperpolarized MRI

**DOI:** 10.1002/mrm.30378

**Published:** 2024-11-17

**Authors:** Kylie Yeung, Kher Lik Ng, Jordan J. McGing, Aaron Axford, Sarah Birkhoelzer, Ayaka Shinozaki, Mattia Ricchi, Noemi Sgambelluri, Fulvio Zaccagna, Rebecca Mills, Andrew J. M. Lewis, Jennifer J. Rayner, Zack Ravetz, Lise Berner, Kenneth Jacob, Anthony McIntyre, Marianne Durrant, Oliver J. Rider, Rolf F. Schulte, Fergus V. Gleeson, Damian J. Tyler, James T. Grist

**Affiliations:** ^1^ Oxford Centre for Clinical Magnetic Resonance Research University of Oxford Oxford UK; ^2^ Department of Oncology University of Oxford Oxford UK; ^3^ Department of Radiology Oxford University Hospitals Oxford UK; ^4^ Oxford Respiratory Service Oxford University Hospitals Oxford UK; ^5^ Department of Physiology, Anatomy, and Genetics University of Oxford Oxford UK; ^6^ Department of Computer Sciences University of Pisa Pisa Italy; ^7^ National Institute of Nuclear Physics (INFN) Division of Bologna Bologna Italy; ^8^ Alma Mater Studorium University of Bologna Bologna Italy; ^9^ Department of Radiology Cambridge University Hospitals Cambridge UK; ^10^ RRPPS University Hospitals Birmingham Birmingham UK; ^11^ GE HealthCare Munich Germany

**Keywords:** B_1_ correction, hyperpolarized ^129^Xe MRI, hyperpolarized ^13^C MRI

## Abstract

**Purpose:**

The purpose of this work is to validate a simple and versatile integrated variable flip angle (VFA) method for mapping B_1_ in hyperpolarized MRI, which can be used to correct signal variations due to coil inhomogeneity.

**Theory and Methods:**

Simulations were run to assess performance of the VFA B_1_ mapping method compared to the currently used constant flip angle (CFA) approach. Simulation results were used to inform the design of VFA sequences, validated in four volunteers for hyperpolarized xenon‐129 imaging of the lungs and another four volunteers for hyperpolarized carbon‐13 imaging of the human brain. B_1_ maps obtained were used to correct transmit and receive inhomogeneity in the images.

**Results:**

Simulations showed improved performance of the VFA approach over the CFA approach with reduced sensitivity to T_1_. For xenon‐129, the B_1_ maps accurately reflected the variation of signal depolarization, but in some cases could not be used to correct for coil receive inhomogeneity due to a lack of transmit‐receive reciprocity resulting from suboptimal coil positioning. For carbon‐13, the B_1_ maps showed good agreement with a separately acquired B_1_ map of a phantom and were effectively used to correct coil‐induced signal inhomogeneity.

**Conclusion:**

A simple, versatile, and effective VFA B_1_ mapping method was implemented and evaluated. Inclusion of the B_1_ mapping method in hyperpolarized imaging studies can enable more robust signal quantification.

## 1 INTRODUCTION

### 1.1 B_1_ mapping

B_1_ mapping is crucial in MRI reconstruction and post‐processing to account for transmit (B_1_^+^) and receive (B_1_^−^) magnetic field inhomogeneities resulting from coil imperfections and loading variations.^1^ B_1_^+^ inhomogeneity refers to the discrepancy between the nominal flip angle (FA), *θ*, and the apparent FA, α, delivered to the imaged volume. B_1_^−^ inhomogeneity refers to the spatial variation of coil receive sensitivity. Together, they form the bias field, and result in non‐physiological signal variations which affect image interpretation and signal quantification.^2^

New B_1_ mapping methods are required for hyperpolarized MRI (hpMRI), a novel contrast mechanism enabled by an artificial, and temporary, increase in the signal obtained from nuclei with naturally low polarization.^3^, ^4^ Unlike conventional MRI, hpMRI signals decay rapidly, meaning standard B_1_ mapping techniques like the double‐angle method (DAM),^5^ the actual FA imaging (AFI)^6^ method, or signal null approaches,^7^ which assume longitudinal magnetization (*M*_z_) recovery, are unsuitable.^8^

### 1.2 Hyperpolarized MRI: Clinical applications

Developing accurate and readily implemented B_1_ mapping methods is an important step toward achieving the promising clinical potential of hpMRI. This study focuses on xenon‐129 (^129^Xe) and carbon‐13 (^13^C), the two most widely researched nuclei due to their advantageous properties for hyperpolarization and their relevance to disease diagnosis and monitoring.^9^, ^10^

^129^Xe hyperpolarized using Spin Exchange Optical Pumping (SEOP)^3^ is a gaseous contrast agent that follows the same pathways as oxygen gas through the lungs after inhalation. Xenon has an apparent T_1_ ranging from 10 to 40 s in the lungs depending on partial oxygen pressure.^11^ It changes frequency as it dissolves into tissue plasma (TP) and again into red blood cells (RBCs),^12^, ^13^ allowing the different phases of ^129^Xe to be imaged. Ratios between gas, tissue, plasma, and red blood cell phase images provide insight into the regional function of the lungs, which has conventionally been one of the most challenging organs to image with MRI.^14^ Recent studies have used hyperpolarized ^129^Xe MRI to evaluate lung diseases such as asthma,^15^ chronic obstructive pulmonary disorder,^16^, ^17^ and long COVID (coronavirus disease 2019).^18^

Carbon‐13 can be hyperpolarized using methods like dissolution dynamic nuclear polarization (dDNP)^4^ or parahydrogen induced polarization (PHIP),^19^ and is used as a metabolic probe after intravenous injection. Although carbon molecules in the body mainly contain the predominant, stable isotope, carbon‐12, which is not MRI‐visible, these molecules can be labeled with carbon‐13 such that they can be hyperpolarized, and their signal can be increased by more than four orders of magnitude above thermal equilibrium. [1‐^13^C]pyruvate is the most widely used molecule due to its involvement in the key reactions of anaerobic glycolysis, and pyruvate transamination and oxidation.^10^ Its conversion into downstream metabolites, lactate and bicarbonate, are of particular interest for studying diseases in a range of organs such as the brain,^20^, ^21^, ^22^, ^23^ heart^24^, ^25^, ^26^, ^27^ and liver,^28^ and can be evaluated by taking the ratios between the metabolite maps and/or modeling kinetics.

### 1.3 Hyperpolarized MRI: Existing B_1_ Mapping Methods

Methods have been explored to map the B_1_ field in hpMRI. B_1_ variation should be accounted for to minimize its downstream effects on quantification, for example in the calculation of hyperpolarized ^129^Xe ventilation defect percentage (VDP) which largely relies on signal intensity differences, and the quantification of metabolite distribution and temporal dynamics in hyperpolarized ^13^C MRI.

The earliest methods were performed outside the clinical imaging setting, for example using a separate measurement coil or a phantom prior to imaging.^29^, ^30^ However, the requirement for additional hardware or hyperpolarized contrast, as well as the need for unrealistic assumptions such as perfect coil performance and minimal patient variability,^31^ render these methods suboptimal for clinical applications. Computational models or filters, like those used in ^1^H imaging,^32^ are often used to compensate for B_1_ in ^129^Xe ventilation imaging.^33^ However, these filters assume the signal variations at low spatial frequencies can be completely attributed to B_1_ inhomogeneity, which is a valid assumption for proton MRI but not hyperpolarized ^129^Xe MRI where gravity and lung elasticity are also contributing factors.^11^

More recently, transmit B_1_ mapping methods have been integrated into imaging to better represent actual scanning conditions and eliminate the need for co‐registration of field maps to images, and these methods can be categorized into phase‐based and magnitude‐based methods. The phase dependence of the MR signal on B_1_^+^ can be extracted using the Bloch‐Siegert method, which has been applied to hyperpolarized ^13^C MRI.^34^, ^35^, ^36^, ^37^ The Bloch‐Siegert method involves inserting an off‐resonance pulse and measuring the B_1_‐dependent phase‐shift it causes. Although relatively robust and invariant to T_1_ decay, the Bloch‐Siegert sequence has a high specific absorption rate and is time limited by the off‐resonance Fermi pulse.^8^ More importantly, integration of a Bloch‐Siegert pulse is not easily implemented in most pulse sequences and requires specialized pulse sequence programming.

Magnitude‐based B_1_ mapping methods have been applied to hpXeMRI, which extract the B_1_ dependence of the hyperpolarized signal by nonlinear least‐squares fitting of sequential pulses. This has been applied in the form of fitting a signal from a train of constant FAs (CFA),^16^, ^38^ and retrospective fitting of signals from successive radial or spiral arms.^11^, ^39^ The main consideration of this approach is that signal decay between successive acquisitions is a result of not only RF depolarization but also the T_1_ decay of the hyperpolarized species. The T_1_ decay is not straightforward to account for, as its value is not well defined and is known to vary with physiology.^8^ Because the imaging time in these previous studies are comparable to the expected T_1_ in hyperpolarized ^129^Xe, a T_1_ value must be assumed.^11^

### 1.4 Variable flip angle B_1_ ^+^ mapping method

This study proposes an optimized approach to the magnitude based B_1_ mapping method,^40^ by varying the FAs used in the FA train and optimizing the total acquisition time to enable more accurate B_1_ estimation. Changing the FA magnitude introduces a term to the signal train that is dependent only on B_1_ and not T_1_, which may allow better fidelity than the CFA approach.^11^, ^16^, ^41^ Keeping total acquisition time short also minimizes T_1_ sensitivity to allow B_1_ variation to be extracted independently. Relative to phase‐based methods like the Bloch–Siegert method, this method is straightforward to implement as it only requires defining scan parameters of a standard pulse sequence instead of designing specialized pulse sequences. Furthermore, the variable FA (VFA) method utilizes the residual magnetization in a hyperpolarized scan, and therefore is minimally disruptive.

Simulations were first used to demonstrate the increased robustness against T_1_ decay of the VFA method compared to the CFA method. Then the VFA method was validated in vivo on a patient‐by‐patient basis for both hyperpolarized ^129^Xe MRI and hyperpolarized ^13^C MRI.

## 2 THEORY

The transmit B_1_^+^ field can be estimated through nonlinear least squares fitting of signal magnitude to a signal model accounting for B_1_ inhomogeneity. In hpMRI, *M*_*z*_ decays, rather than recovers, to equilibrium over time and with the application of every RF pulse.^42^ T_1_ decay is characterized by *e* ^−*t*/*T1*^, which approaches 1 as *t* is shortened, and decreases exponentially as *t* is lengthened. The equations describing *M*_*z*_ and *M*_*xy*_ are expressed as:

(1) $$\begin{array}{ll} M_{z}\left(t\right) & =\left(M_{0}+\left(M_{z,\mathrm{HP}}-M_{0}\right)\exp\left(-\frac{t}{T_{1}}\right)\right)\\ \prod_{j=1}^{n}\cos\left(\alpha_{j}\right) & ≅M_{z,\mathrm{HP}}\;\exp\left(-\frac{t}{T_{1}}\right)\prod_{j=1}^{n}\cos\left(\alpha_{j}\right) \end{array}$$

and

(2) $$M_{\mathrm{xy}}\left(t\right)=M_{z,\mathrm{HP}}^{\cdot }\;\exp\left(-\frac{t}{T_{1}}\right)\cdot \sin\left(\alpha_{n}\right)\prod_{j=1}^{n-1}\cos\left(\alpha_{j}\right),$$

where $M_{0}$ is the thermal equilibrium magnetization, $M_{z,\mathrm{HP}}$ is the initial hyperpolarized magnetization, and *n* is the number of RF excitations.^43^ *M*_*xy*_ and the hyperpolarized signal *S* can be expressed with respect to ΔB_1_^+^ and ΔB_1_^−^ as:

(3) $$\begin{array}{ll} M_{\mathrm{xy}}\left(x\right) & =M_{z,\mathrm{HP}}\left(x\right)\cdot \exp\left(-\frac{\mathrm{TR}\cdot n}{T_{1}\left(x\right)}\right)\\ & \;\cdot \sin\left(∆{B_{1}}^{+}\left(x\right)\cdot \theta_{n}\right)\cdot \prod_{j=1}^{n-1}\cos\left(∆{B_{1}}^{+}\left(x\right)\cdot \theta_{j}\right) \end{array}$$

and

(4) $$S\left(x\right)=∆{B_{1}}^{-}\left(x\right)\cdot M_{\mathrm{xy}}\left(x\right).$$

A Δ*B*_1_^+^ smaller than 1 indicates under‐flipping and larger than 1 indicates over‐flipping. By predefining a train of FAs and using a known TR, the unknown $∆{B_{1}}^{+}\left(x\right)$ and $M_{z,\mathrm{HP}}\left(x\right)$ can be solved voxel‐wise as a nonlinear least‐squares problem.

The train of FAs should be selected with a large enough increment to fit for ΔB_1_, while efficiently utilizing the limited magnetization. Different ordering schemes of the FAs are viable, for example [3 × 10°, 3 × 20°, 3 × 30°] or [3 × (10°, 20°, 30°)]. Each have their respective risks and benefits: the former risks the signal running out or patient movement before the end of the acquisition, but the latter risks depleting the signal too quickly by larger RF excitations toward the start.

As it is challenging to separately measure transmit and receive profiles of a transmit/receive (Tx/Rx) coil, reciprocity is often assumed in currently existing B_1_ correction methods.^11^, ^41^, ^44^ The reciprocity principle refers to the symmetry between the transmission and reception of RF signals by the coil, where a region of the coil with higher transmit efficiency would have proportionally higher receive sensitivity and vice versa.^45^ This can be intuitively understood by imagining a loop coil used either to induce a magnetic field or to measure the change in magnetic flux of a moving magnet: the larger the distance between them, the smaller the induced magnetic field or the detected current. This principle, however, is based upon the assumption that there is no geometric coupling between different coil elements.

## 3 METHODS

### 3.1 Simulations

Evaluation of different magnitude based B_1_ estimation approaches was performed by simulating hyperpolarized MR signals over a range of acquisition conditions, corrupting the signals by random noise, then testing the estimation accuracy of the CFA versus the VFA approach.

MR signals were simulated in MATLAB (2023b, The Mathworks, Cambridge, MA) using Eq. (3). Signals were simulated with a T_1_ = 23 s^46,47^ across a range of total acquisition times (0.1 of T_1_ to 1.3 of T_1_), and across a range of initial FAs (10–60°). Varying the total acquisition time and TRs relative to the simulated T_1_ allows the interpretation of the simulation results to be applicable to different true T_1_ values. The FA was set to repeat 3, 9, or 18 times (*N*_FA_) within the defined total acquisition time. In the CFA case, the FA was the same for every repetition, whereas the VFA case had three different FAs incremented at 10°. For example, the VFA train with initial FA 10° and *N*_FA_ = 9 would be [10, 10, 10, 20, 20, 20, 30, 30, 30]^o^. Each simulation was run 1000 times and corrupted with additive random noise at 1% of *M*_z,HP_, chosen to mimic the SNR level of hyperpolarized imaging experiments (using a 10° FA, the resulting SNR would be $\left(\sin10^{o}\times M_{z,\mathrm{HP}}\right)/\left(0.01\times M_{z,\mathrm{HP}}\right)≅17$). The SNR‐corrupted signals were then fit using a nonlinear least‐squares fitting routine (*lsqcurvefit*) to estimate ΔB_1_ and *M*_z,HP_.

To investigate the T_1_ sensitivity of the CFA method versus the VFA method, four different approaches to T_1_ assumption were tested: (1) underestimating T_1_, (2) assuming the correct T_1_, (3) overestimating T_1_, and (4) not assuming but instead fitting for the unknown T_1_. An “underestimation” of T_1_ was chosen to be 10 s, and an “overestimation” 40 s. The fourth approach was not applied to the *N*_FA_ = 3 case because there are not enough data points to fit for three unknowns (B_1_, *M*_z,HP_, and T_1_).

Estimation accuracy was evaluated by the mean percentage error between estimated ΔB_1_ values and the ground truth simulated values.

### 3.2 Data acquisition: ^129^Xe imaging of the lung and ^129^Xe coil characterization

**FIGURE 1:**
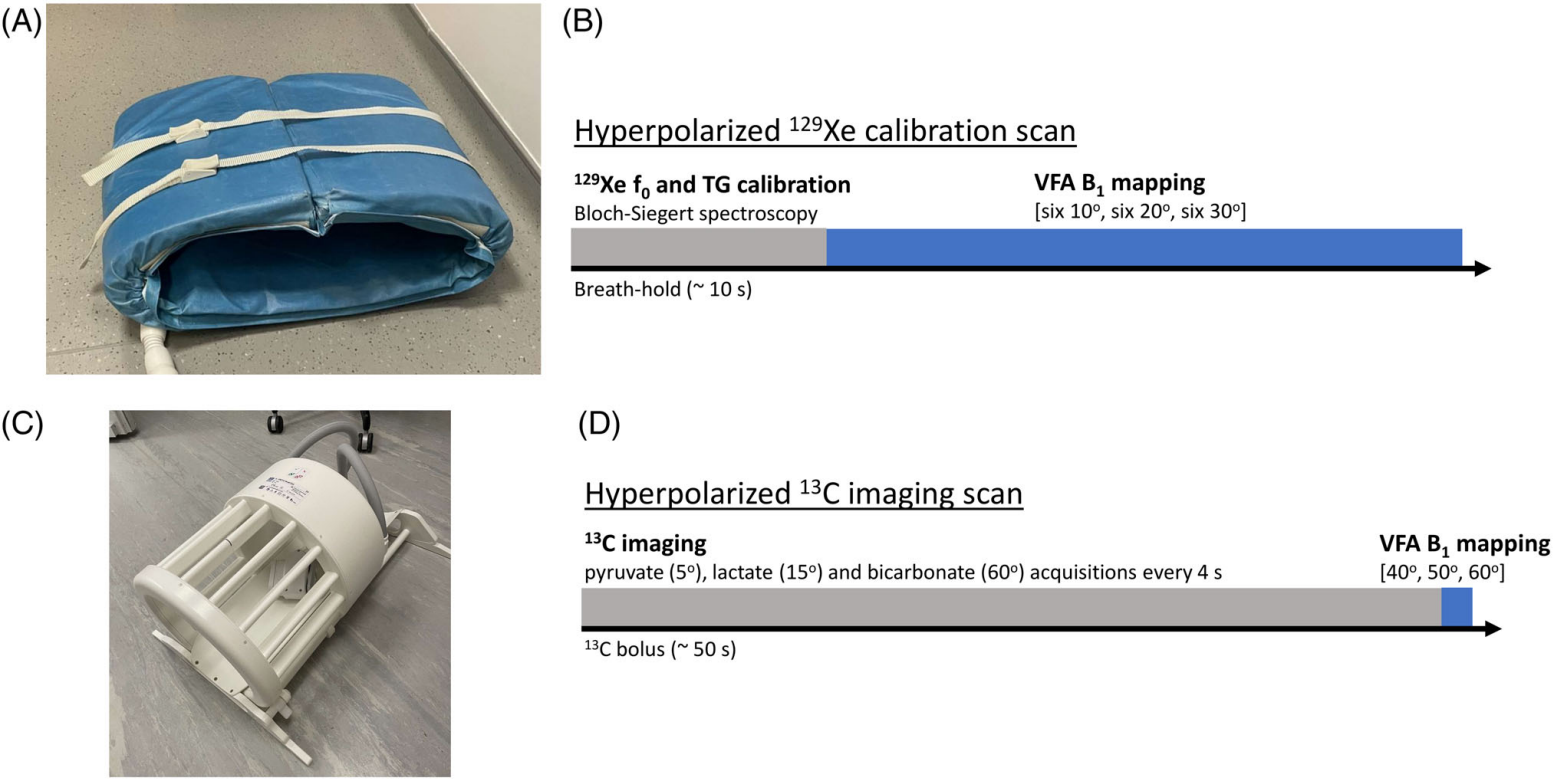
Coils and VFA acquisition schemes used for in vivo B_1_ mapping with hyperpolarized ^129^ Xe and ^13^C. (A) Flexible ^129^Xe wrap‐around coil. (B) Schematic showing integration of VFA B_1_ mapping acquisition into ^129^Xe calibration scan. (C) Dual‐tuned ^1^H/^13^C birdcage head coil. (D) Schematic showing integration of VFA B_1_ mapping acquisition into ^13^C imaging scan.

The VFA method was tested in participants who had fully recovered from COVID‐19 (three female, one male; mean age = 37 ± 11 y), as part of a prospective long‐COVID study approved by the South Central ‐ Oxford Research Ethics Committee. All participants gave written informed consent. Participants were imaged using a 3T GE Premier MRI scanner (GE Healthcare, Milwaukee, USA) with a flexible Tx/Rx ^129^Xe wrap‐around coil (PulseTeq, Cobham, UK) (Figure 1A). The coil consists of four loop elements, two posterior, two anterior, with diagonal elements connected to form two Tx/Rx channels, whose signals are combined in hardware before output. A phantom large enough to fill the field of view was unavailable. Instead, coil element performance was characterized using a vector network analyzer (ZNLE3, Rhode and Schwarz, Munich, Germany) to measure the impedance matching (S_11_ and S_22_) for each of the channels on two participants.

The VFA B_1_ mapping sequence was integrated into the routine Bloch‐Siegert calibration scan with a 10% dose of ^129^Xe prior to dissolved phase imaging. Enriched ^129^Xe was polarized for ˜10 min in a commercial polarizer (Polarean, NC, USA). Participants inhaled 1 L of hyperpolarized gas containing a mixture of xenon and nitrogen (0.1:0.9 L, respectively). The Bloch‐Siegert scan was a 2‐s spectroscopic acquisition^34^ (1024 points, Number of scans = 32, TR = 75 ms, hard pulse excitation, FA = 10°, bandwidth = 20 kHz), using a center frequency (f_0_) estimated from the water peak.^46^ Though the Bloch‐Siegert scan estimates optimal TG, the TG was not updated for the VFA scan which was performed in the remainder of the breath‐hold. A TG value of 200 was used for all participants because experience with this coil shows 200 is the required minimum for most participants, and the software imposes a maximum limit of 200. Immediately after the Bloch‐Siegert Scan, the VFA multi‐slice spiral sequence was played (16 × 16 acquisition matrix, 12 15 mm slices, FOV = 400 × 400 mm, 1.8 ms partially self‐refocused excitation pulse, 32 × 32 reconstruction matrix, TR = 230 ms, total scan time = ˜4 s, bandwidth = 250 kHz), using six 10°, six 20°, and six 30° acquisitions (Figure 1B), implemented using the *MNS Research Pack* (GE Healthcare, Milwaukee, USA). For the VFA fitting, an estimated T_1_ value of 23 s^47^, ^48^ was used.

### 3.3 Data acquisition: ^13^C imaging of the brain and ^1^H/ ^13^C coil characterization

The VFA sequence was validated in five human brain scans of four participants (all male; ages 62.5 ± 3.9 y). The study was approved by a local ethics committee (South Central, 20/SC/0441) and participants provided written informed consent. The participants were scanned in a 3T Premier MRI scanner, different from the one used for ^129^Xe imaging, and using a dual‐tuned ^1^H/^13^C head coil (Rapid Biomedical, Rimpar, Germany) (Figure 1C). For the ^1^H/^13^C birdcage head coil, a separate B_1_ map was acquired using CSI measurements of a spherical silicone oil phantom (12 × 12 × 7 voxels, 512 points, TR = 4 s) at seven incrementing FAs [40, 60, 80, 90, 110, 150, 180]^o^. The data were then fitted voxel‐wise to the following equation:

(5) $$S=M_{0}\sin\left(∆B_{1}\cdot \theta \right).$$

The hyperpolarized ^13^C pyruvate was prepared by polarizing 1.47 g of [1‐^13^C]pyruvic acid (Merck, New Jersey, USA) with 15 mM AH111501 (Syncom Healthcare, Groningen, Netherlands) for approximately 4 h in a SPINLab hyperpolarizer (GE Healthcare, Milwaukee, USA),^49^ followed by dissolution with sodium hydroxide buffer. After quality control checks, the ^13^C bolus was injected at 5 mL/s intravenously based on the participant weight (0.4 mL/kg), followed by a 20 mL saline flush. The time from dissolution to injection was recorded. *f*_*0*_ was adjusted relative to the proton frequency and optimal TG was determined using a prior non‐slice‐selective Bloch‐Siegert acquisition of a ^13^C‐labeled urea phantom attached to one of the rungs of the birdcage coil. This optimal TG was used for the subsequent imaging scan.

The imaging sequence was a multi‐slice, spectral‐spatial spiral sequence^50^ (16 × 16 acquisition matrix, FOV = 240 × 240 mm, eight 20 mm slices, 32 × 32 reconstruction matrix, TR = 490 ms) with pyruvate, lactate, and bicarbonate acquisitions every 4 s at FAs of 5°, 15°, and 60°, respectively.

The VFA B_1_ mapping sequence for hyperpolarized ^13^C was integrated into the imaging sequence itself and optimized for minimum acquisition time due to the quickly flowing ^13^C bolus through the brain. The B_1_ map was acquired at the pyruvate frequency (˜45 s after the start of bolus injection) using three FAs [40, 50, 60]° (Figure 1D). The 490 ms TR was determined by the gradient and slew rate limitations of the sequence and coil, resulting in a 1.5 s acquisition time for the B_1_ map. For the VFA fitting, an estimated T_1_ value of 56 s^51^ was used, which is roughly within the range of literature values. Given that the entire acquisition was very short relative to literature values of T_1_, a slight deviation in estimated T_1_ would not affect the fitted B_1_ value.

### 3.4 Signal processing and image correction

Using the defined TRs and FA trains for the ^129^Xe and ^13^C acquisitions, respectively, images were fit voxel‐wise to Equation (3) using the *curve_fit* function in the SciPy toolbox in Python 3.11.^52^ Goodness‐of‐fit was evaluated using the normalized RMS error (NRMSE). Polynomial fitting and extrapolation^53^ (second‐order, kernel size 4) was used to smooth masked voxels under the assumption that B_1_ inhomogeneity has a slow spatial variation.

For the ^129^Xe images, B_1_ transmit correction was applied to acquisitions at each time point as follows:

(6) $$I_{n}^{\prime }=I_{n}\cdot \frac{1}{\sin\left(∆{B_{1}}^{+}\left(x\right)\cdot \theta_{n}\right)\cdot \prod_{j=1}^{n-1}\cos\left(∆{B_{1}}^{+}\left(x\right)\cdot \theta_{j}\right)}.$$

For the ^13^C images, each of the metabolite maps were first divided by the sine of their respective acquisition FA. ΔB_1_^+^ inhomogeneity in the summed time‐series ^13^C signals was corrected for using the equation below:

(7) $$I_{\text{metabolite}}^{\prime }=I_{\text{metabolite}}\cdot \frac{\sin{\mathrm{FA}}_{\text{metabolite}}}{\sin\left(∆B_{1}\cdot {\mathrm{FA}}_{\text{metabolite}}\right)}.$$

Under the assumption of transmit/receive reciprocity, ΔB_1_^−^ was then accounted for by dividing the ΔB_1_^+^‐corrected image by the ΔB_1_ measured using thes VFA method.

Images were masked individually by manual selection of an appropriate signal magnitude threshold, to exclude outer voxels where hyperpolarized signal is not present. The signal ratio for each voxel after and before B_1_ correction was calculated.

## 4 RESULTS

### 4.1 Simulation

**FIGURE 2:**
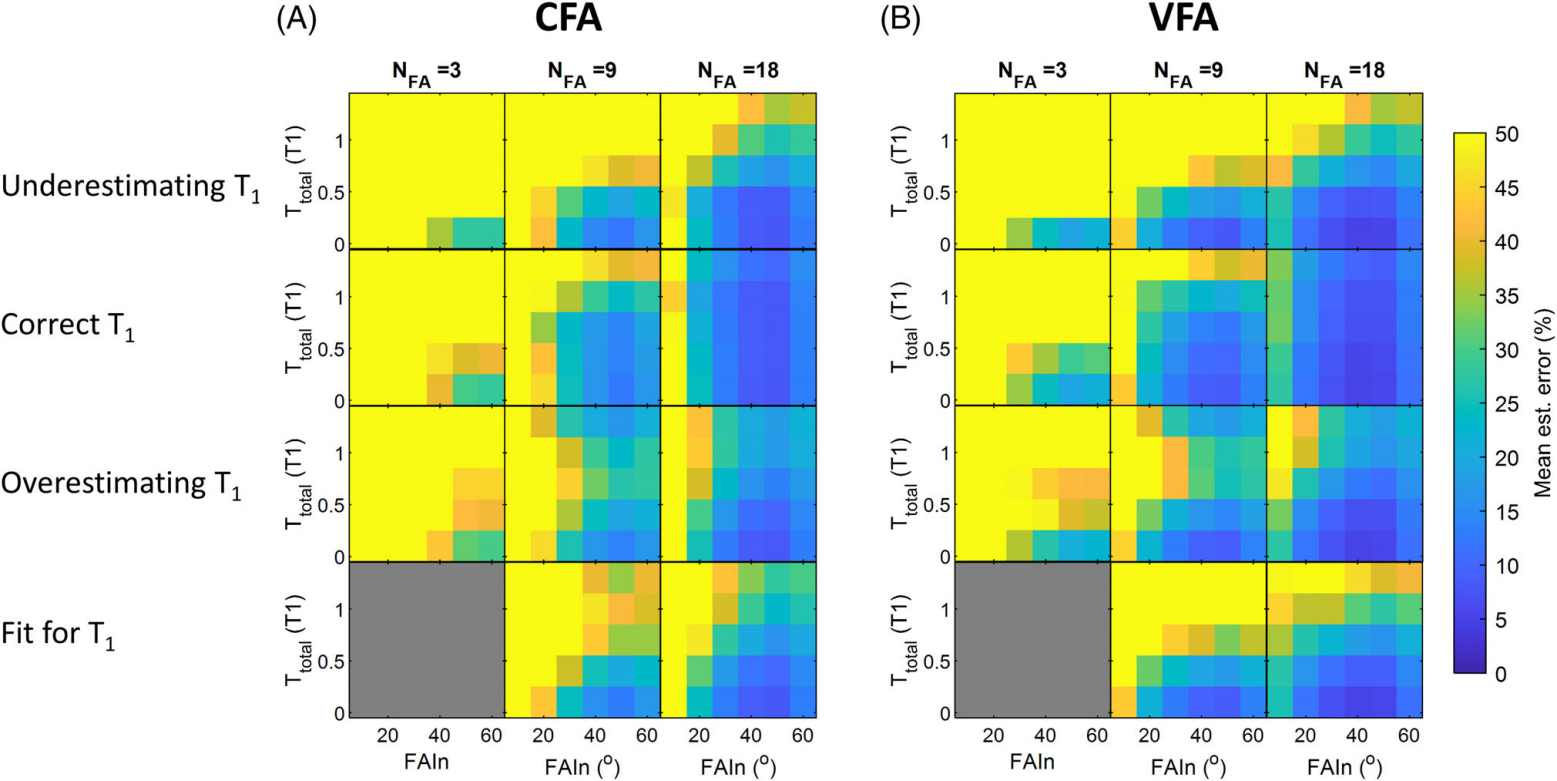
Simulation results comparing the performance of the (A) CFA scheme to the (B) VFA scheme, across a range of initial FAs (FAIn), total acquisition times (*T*_tot_, expressed as ratio to the simulated T_1_), and number of FAs (*N*_FA_). Four different approaches to T_1_ were investigated: Assuming a lower T_1_, assuming the correct T_1_, assuming a higher T_1_, and including the T_1_ as a fitting term. Reduced estimation errors using the VFA method demonstrated.

Mean percentage errors of the simulated estimation approaches are shown in Figure 2, with the color‐bar scaled to 50% to disregard the noisiest cases that produced unrealistic estimation results. When T_1_ is assumed, the VFA method outperformed the CFA method in almost all cases, regardless of whether the T_1_ is underestimated, correctly estimated, or overestimated. Even though, given the same initial FA, the VFA method used higher FAs and depolarized the signal quicker than the CFA method, it still achieved a lower mean percentage error in 135 out of 145 cases. In their respective best cases, the VFA method was able to achieve a mean percentage error of 2.7%, whereas CFA was able to achieve 5.9%.

The lowest mean estimation errors were achieved when total acquisition time was minimized and when the number of FA repetitions (*N*_FA_) were increased, achieving ˜3% mean estimation error with initial FA of 40° and *N*_FA_ = 18 using the VFA method. Fitting for T_1_ does not improve B_1_ estimation. For the same FAs and TRs used, more repetitions results in a better fit; for example, at the correctly estimated T_1_, using a 40° initial FA gives an error of ˜30% with *N*_FA_ = 3 and ˜6% with *N*_*FA*_ = 18 in the CFA case, and an error of over 14% with *N*_FA_ = 3 and under 3% with *N*_*FA*_ = 18 in the VFA case. Using higher FAs generally improves estimation, until the FA becomes too high and signal is depleted; for example, in the *N*_FA_ = 3 case, estimation error decreases with increasing initial FA from over 140% to under 25% in the CFA case and from over 60% to under 16% in the VFA case. With *N*_FA_ = 18 in the VFA case, the estimation error decreases from 13.6% at an initial FA of 10° to 2.8% at initial FA of 40°, then increases to 10.3% at initial FA of 60°.

### 4.2 Xenon‐129 imaging

ΔB_1_^+^ maps were acquired for the four participants. For each participant, over 90% of the fitted voxels had NRMSE of less than 0.17. The mean B_1_ values measured also correspond well to the TG measured during the Bloch‐Siegert acquisition (see Data S1).

**FIGURE 3:**
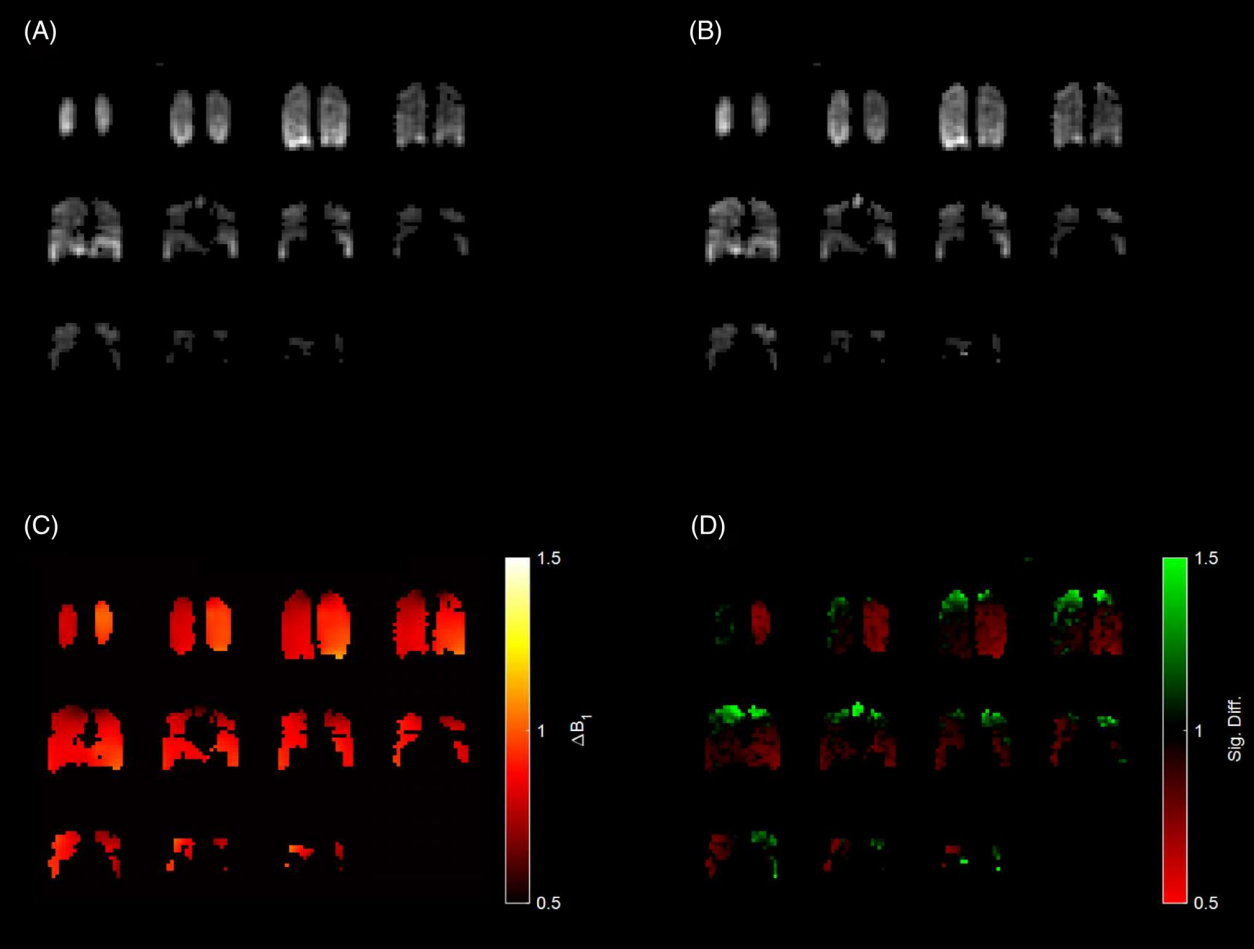
Example of B_1_ transmit and receive correction in one of the participants imaged with hyperpolarized ^129^Xe. (A) The summed signal before B_1_ correction. (B) The summed signal after B_1_ correction. (C) The fitted B_1_ map used for correction. (D) The signal difference after B_1_ correction (i.e., dividing the corrected image over the original image). Slices are shown from the posterior to the anterior of the patient. Signal drop‐off toward the top of the lung can be corrected.

**FIGURE 4:**
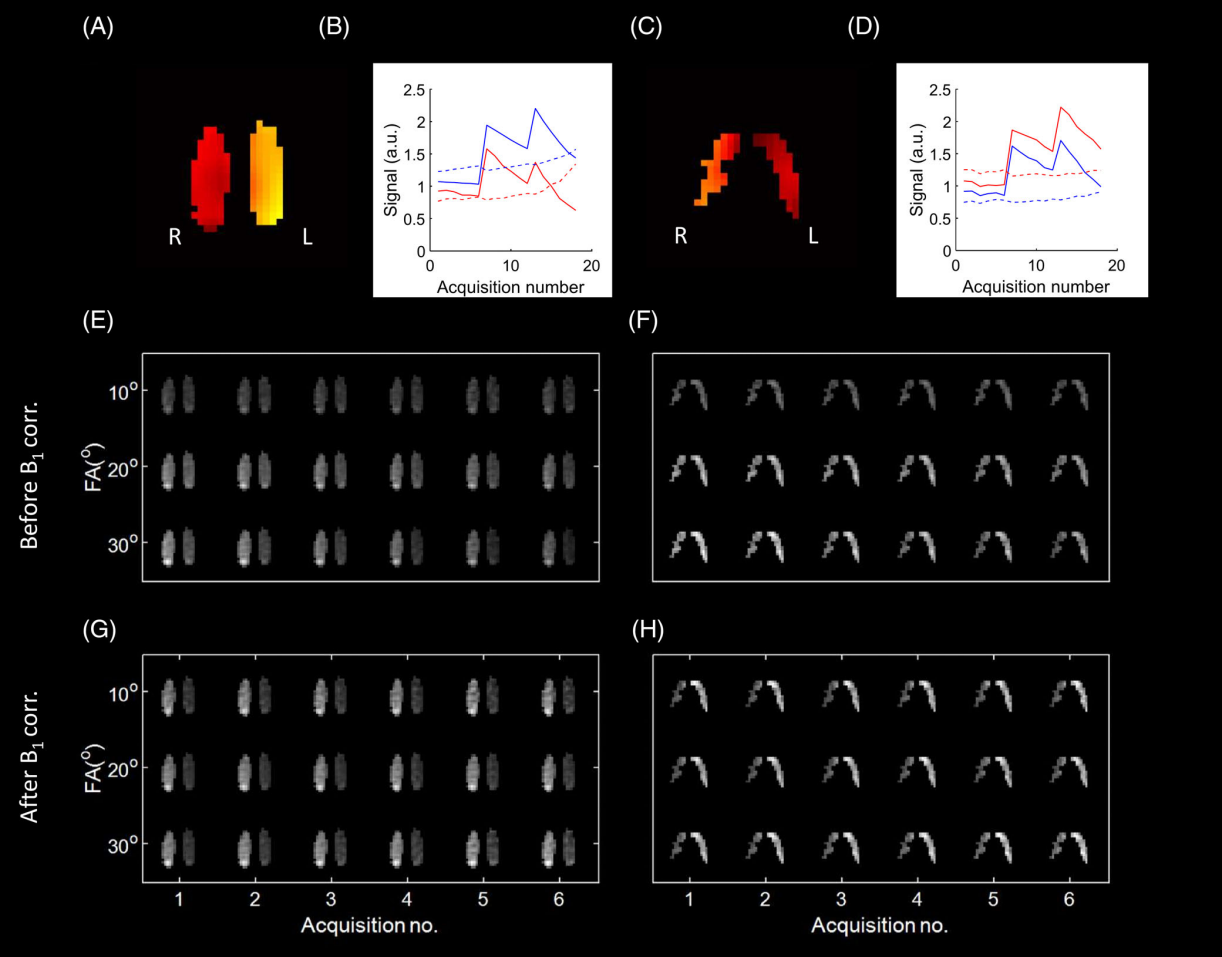
B_1_ correction in hyperpolarized ^129^Xe imaging of a relatively light participant. The B_1_ profiles and signal evolution over the 18 acquisitions in the VFA sequence are shown for (A, B) slice 2 (posterior) and (C, D)slice 9 (anterior). (In B and D, blue indicates mean signal from the right lung, and red from the left lung. Solid line shows signal before applying B_1_^+^ correction, dotted line shows signal after applying B_1_^+^ correction. Signals are normalized to the mean value of the initial acquisition before and after correction, respectively.) (E, F) Signal evolution over time from these two slices, respectively. G, H The same signal evolution over time after applying B_1_^+^ correction.

In the example of one participant, applying the B_1_ transmit and receive correction can enhance the signal toward regions where a signal drop‐off is expected, that is, toward the top of the lungs nearing the edge of the coil (Figure 3). However, B_1_ correction did not produce sensible results for a smaller patient of 60 kg, likely because the flexible four‐loop coil did not maintain Tx/Rx reciprocity. Signal evolution over time shows that the transmit B_1_ field can be mapped (Figure 4). Regions that depolarize quicker over the course of the acquisition indeed correspond to higher ΔB_1_^+^ values. For example, in slice 2 (Figure 4E), the signal in the left lung is 13% lower than the right lung in the initial acquisition and becomes 60% lower in the final image, and thus the left lung was mapped to have a relatively high ΔB_1_^+^ value compared to the right lung. Applying ΔB_1_^+^ correction (Figure 4G) allows the signal ratio between the left and right lung to be maintained from the initial acquisition to the last. However, the signal difference remaining after ΔB_1_^+^ correction does not match what is expected if the ΔB_1_^+^ and ΔB_1_^−^ profiles were reciprocal, which is that the side of the lung with higher ΔB_1_^+^ should have a higher signal after transmit correction.

Further testing with the ^129^Xe coil suggests that the lack of Tx/Rx reciprocity in some cases could be attributed to geometric coupling of the coil elements when the coil is not optimally positioned around the wearer. Since the wrap‐around coil must accommodate a range of different body shapes, there is an inevitable compromise in performance across subjects. Optimal positioning can be achieved for some patients but may be a challenge for others. It was found with vector network analyzer measurements that the two channels in the coil had a −14 dB isolation when worn by a participant who was able to maintain the distance between the coil elements and had a higher filling factor, and a −6 dB isolation when worn by a smaller participant with a lower filling factor where the coil elements became closer together.

### 4.3 Carbon‐13 imaging

**FIGURE 5:**
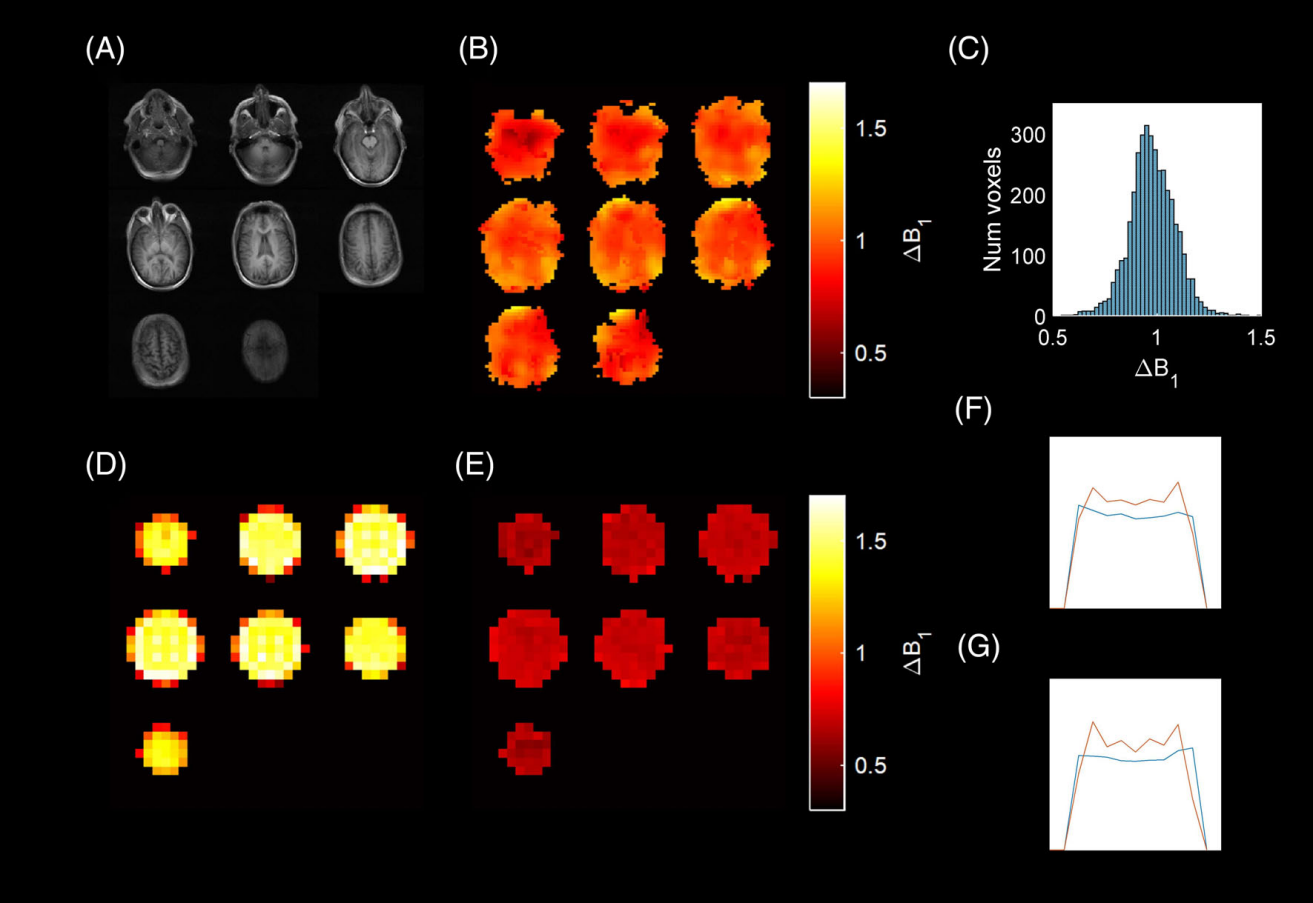
From one of the participants imaged with hyperpolarized ^13^C: (A) anatomical ^1^H T_1_‐weighted image, (B) corresponding B_1_ map acquired with VFA method, (C) histogram showing distribution of fitted B_1_ values. From CSI acquisition of a silicone oil phantom: (D) *M*_0_ map, (E) B_1_ map, and (F, G) slice profiles of *M*_0_ (red) and B_1_ (blue) through the center of slices 4 and 5 in the phantom, respectively.

Four participants were successfully imaged, with one of the participants scanned twice. The time between dissolution and injection was 70 ± 11 s. Mean ΔB_1_ was 0.98 ± 0.11, 0.92 ± 0 0.12, 0.90 ± 0.11, respectively, for the patients scanned once, and 0.95 ± 0.12 and 0.93 ± 0.13 for the two scans of the patient scanned twice. A total of 90% of masked voxels had NRMSE of 0.21 or less. The ^1^H T_1_ weighted image, ^13^C B_1_ map, and histogram of B_1_ values of one of the participants is shown in Figure 5A–C. Comparison with the TG measured with the urea phantom can be found in the Data S1.

The in vivo B_1_ maps matched spatially with the coil profile measured using CSI (Figure 5E), and as expected for a quadrature birdcage coil.^54^ The mean ΔB_1_ from the coil profile measured with CSI was lower (0.70 ± 0.04), which could be attributed to sub‐optimal loading and which agrees with previous characterization of the coil.^55^ A similar U‐shaped profile in both *M*_0_ and B_1_ maps from the phantom CSI acquisition suggests Tx/Rx reciprocity (Figure 5D–G).

**FIGURE 6:**
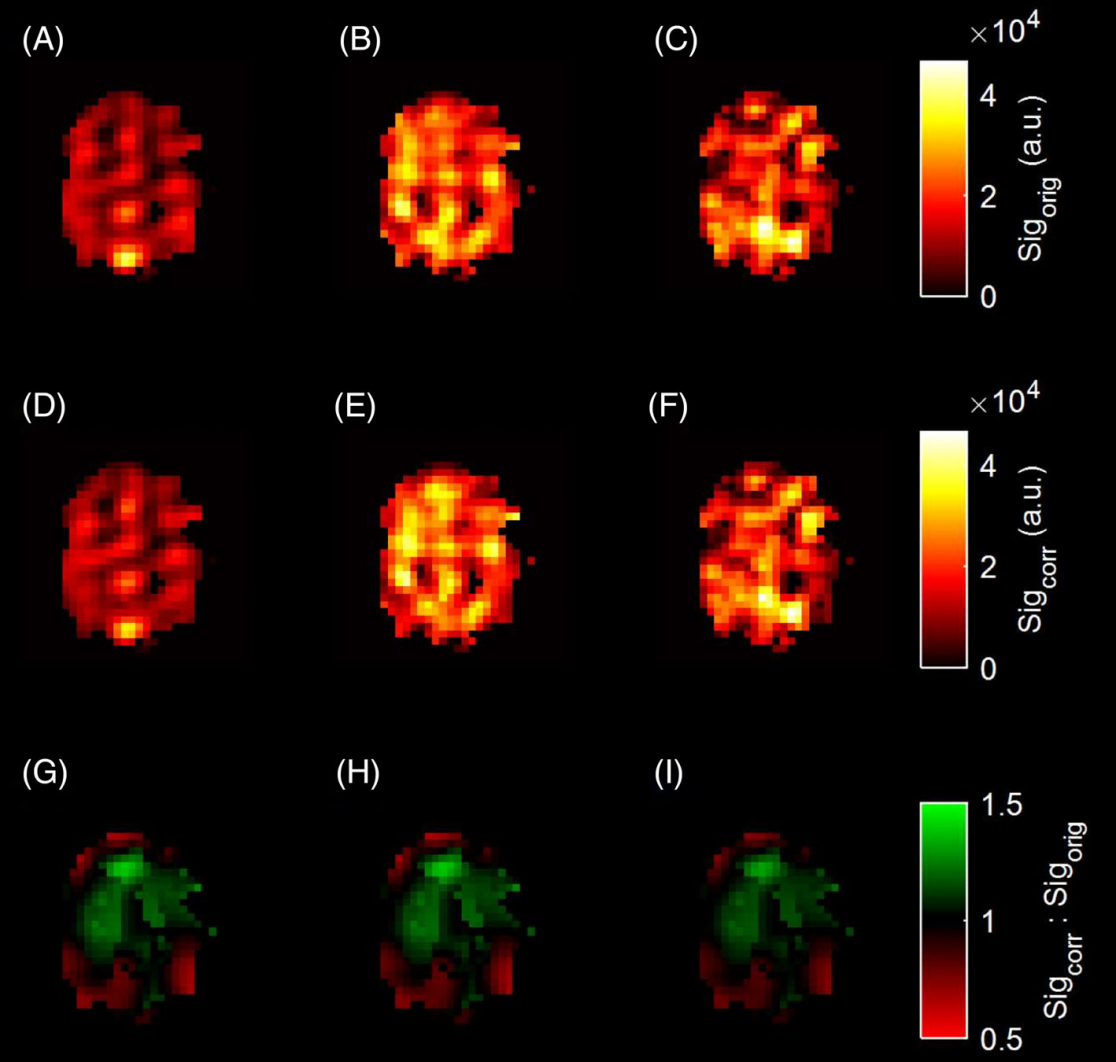
B_1_ correction applied to hyperpolarized ^13^C brain scan. (A) Pyruvate, (B) lactate, and (C) bicarbonate images before applying B_1_ correction. (D) Pyruvate, (E) lactate, and (F) bicarbonate images after applying B_1_ correction. (G–I) Ratio of corrected image over uncorrected image for each metabolite.

Pre‐ and post‐B_1_ corrected metabolite maps (pyruvate, lactate, and bicarbonate) from the central slice of Participant 1 are shown in Figure 6. The mean percentage difference in signal after B_1_ correction is 20 ± 30% in the metabolite maps, demonstrating a consistent under‐flipping in the acquisition due to the urea phantom being close to the coil rungs when calibrating TG.

## 5 DISCUSSION

Simulations evaluated the performance of a VFA B_1_ mapping approach against the current CFA approach, especially with respect to their sensitivities to T_1_ decay. By varying the FAs, simulations show the VFA method increases robustness against the misestimation of T_1_. The simulations underlined that minimizing total acquisition time reduces T_1_ sensitivity and is crucial in achieving good B_1_ estimation. However, acquiring more data points, that is, more repetitions of FAs, also increased estimation accuracy. There is a trade‐off between acquiring more data points and minimizing total acquisition time, which must be optimized alongside the minimum achievable TR of the sequence based on gradient and slew rate limitations, as well as the physiological limitations of available acquisition time. When the acquisition time becomes too long and FAs used are too high, the signal depletes quickly, resulting in inaccurate B_1_ estimation from noisy data.

Simulation results were subsequently used to inform the design of optimized VFA sequences for imaging situations with different physiological constraints. The main physiological constraint is the flow and depolarization of the hyperpolarized probe, which limits acquisition time. The time available for VFA mapping in the ^129^Xe scan was a breath‐hold, allowing a longer, lower‐flip‐angle sequence to be used. In contrast, a shorter, higher‐flip‐angle VFA sequence was needed for ^13^C due to the high pyruvate bolus flow rate.

This work demonstrated the versatility and effectiveness of the VFA method in these two imaging contexts with different hyperpolarized nuclei, organs of interest, and coil architecture. Low NRMSEs show that the signal model was well‐fit and is a good description of experimental results. The acquired B_1_ maps are inherently co‐registered and patient‐specific, so can be used to correct B_1_ inhomogeneity on a person‐by‐person basis. The optimal TG can be first estimated either using the urea phantom as was done here, or from corresponding proton and sodium measurements,^56^ then spatial B_1_ variation can be mapped using the VFA method. The VFA mapping method also has the advantage of being readily implemented into existing hyperpolarized imaging routines. It only requires adjusting the parameters of a standard pulse sequence, which is less involved than designing a specific pulse sequence as used in the Bloch‐Siegert method. Furthermore, the VFA method efficiently uses the limited polarization in hyperpolarized MRI. In the carbon‐13 case, residual magnetization at the end of the imaging sequence was used up, and in the xenon‐129 case, only the signal remaining from a small 10% calibration dose was used.

The B_1_ maps acquired in vivo with the ^129^Xe wrap‐around coil allow B_1_^+^_/_ B_1_^−^ image correction to be applied in some cases. The B_1_ maps accurately describe the signal depolarization over repeated acquisitions, and match with what is known of the coil architecture. However, the VFA acquisitions also highlighted that B_1_^+^_/_ B_1_^−^ image correction is applicable only for situations in which Tx/Rx reciprocity can be assumed, as many previous papers have noted.^11^, ^41^, ^44^ Since quadrature performance is heavily dependent upon the geometrical symmetry of the coil, mismatched elements or geometric asymmetry could contribute to degraded quadrature, and lead to a subsequent lack of reciprocity. Suboptimal coil fit on the wearer, as well as potential shifting of coil elements over repeated use can contribute to the lack of Tx/Rx reciprocity.^57^ This work cautions against simply assuming Tx/Rx reciprocity when applying B_1_ correction in all cases.

To separately map the receive B_1_^−^ is challenging. Although the fitting algorithm allows the separation of the *M*_*z*,HP_ map from the B_1_^+^ map, the confounding effect of uneven gas distribution cannot be removed from the *M*_*z*,HP_ map to obtain the B_1_^−^ map. Nevertheless, the transmit B_1_ map provides valuable information for the correction of time‐series acquisitions, for example dynamic lung imaging where changes throughout the breathing cycle are used to better understand pulmonary physiology.^38^

Where Tx/Rx profiles are reciprocal, the VFA mapping method enables B_1_^+^_/_ B_1_^−^ image correction. The similarity in the *M*_0_ and the B_1_ profiles of the ^1^H/^13^C head coil suggests that Tx/Rx reciprocity was valid in this case, thus allowing image correction under the reciprocity assumption. Applying the B_1_ correction in the ^13^C brain images accounts and corrects for the higher B_1_ toward the anterior and around the perimeter of a participant's brain, which is closer to the coil surface with the participant in a supine position. Given that the urea phantom was attached to the rungs of the coil when TG calibration was performed, B_1_ correction accounts for under‐flipping toward the center of the brain. The VFA method was used to retrospectively correct for both the transmit and receive profiles of the coil, which enables more accurate quantification of metabolites and kinetic modeling.

B_1_^+^_/_ B_1_^−^ image correction is more important for images of one metabolite than it is for ratio maps, and depends on the difference between the nominal FAs used for each metabolite image. For example, with 20% over‐flipping, that is, ΔB_1_ of 1.2, the changes after and before B_1_ correction in the 5° pyruvate, 15° lactate, and 60° bicarbonate images would be −30.5%, −30.2%, and −24%, respectively, but would be 0.4%, 9.2%, and 8.7% in the lactate‐to‐pyruvate, bicarbonate‐to‐pyruvate, and bicarbonate‐to‐lactate images, respectively. Thus, B_1_ mapping is more important for evaluating absolute signal^58^, ^59^ or metabolite distribution and kinetics in hyperpolarized ^13^C MRI.^60^, ^61^ Similarly, B_1_ mapping is less important for ratio maps between different phases in xenon‐129 images, but would be important when a single phase is looked at, for example, interpretation of ventilation defects^62^ or to correct ratios between the gas and RBC/TP peaks, where a different FA may be used to excite resonances.

### 5.1 Limitations and future work

The major limitation of this B_1_ mapping method, and other hyperpolarized B_1_ mapping methods to date, is that patient‐specific B_1_^−^ correction can only be performed by assuming Tx/Rx reciprocity. For a rigid birdcage coil like the one used for ^13^C imaging in this work, the coil behaves linearly, meaning there is good quadrature and little reflected power, thus Tx/Rx reciprocity can be assumed with reasonable confidence. But it is challenging to separately measure the receive profile in vivo when a flexible coil like the ^129^Xe wrap‐around coil is used. A large uniform ^129^Xe gas phantom, if available, would be useful for separate characterization of the transmit and receive profiles of the flexible wrap‐around coil, but would still not be able to account for the potentially large variability in patient loading. Alternatively, using a separate birdcage Tx/Rx coil could be useful for measuring the receive profile in hyperpolarized gas MRI in vivo. For transmit separated multi‐receive coils, this approach would not be applicable.

There is still room for further optimization of the VFA mapping method, and any optimal approach will depend on the constraints of each specific imaging experiment. Reducing the resolution of the B_1_ map could reduce the minimum achievable TR of each acquisition and further reduce the acquisition time and minimize flow effects. The current work only involved single channel data, but the VFA method applied to multi‐channel coil data could be used for optimizing coil combination.

## 6 CONCLUSIONS

A VFA method to map B_1_ in hyperpolarized MRI was proposed and validated. It was shown via simulations that the VFA method achieved lower errors relative to the conventionally used CFA approach. B_1_ maps acquired using the VFA method were able to correct for B_1_^+^_/_ B_1_^−^ inhomogeneity in ^129^Xe lung images and ^13^C brain images, accounting for under‐flipping and reduced coil sensitivity in regions further from the coil surface. This simple, versatile, and effective B_1_ mapping method has the advantages of not requiring additional doses of the hyperpolarized probe, not requiring specialized pulse sequence programming, and producing field maps that are patient‐specific and inherently co‐registered. Future implementation of reliable B_1_ mapping methods such as the VFA method proposed here will enable more robust quantification of physiological biomarkers in hyperpolarized MRI and enhance its clinical value.

## CONFLICT OF INTEREST STATEMENT

RFS is an employee of GE HealthCare. The hyperpolarized Xenon lung datasets included are from a study in collaboration with the University of Sheffield (EXPLAIN).

## Supporting information


**Figure S1:** T1 estimation errors using the CFA approach with fifteen 10° acquisitions (top) and VFA approach with five 10°, five 20°, and five 30° acquisitions (bottom). T_1_ was varied with the TR kept at 0.23 s (left), and TR was varied with the T_1_ kept at 23 s.
**Figure S2:** Signal train from a hyperpolarized carbon‐13 phantom using a short TR (left) and a long TR (right), showing that T_1_ agrees with literature value^51^ if TR and total acquisition time is long enough.
**Figure S3:** B_1_ maps from five hyperpolarized carbon‐13 VFA scans, showing mean ΔB_1_ values close to 1.
**Figure S4:** B_1_ maps from the hyperpolarized Xenon‐129 VFA scans (top, middle rows), scatter plot of mean ΔB_1_ against the TG used divided by the optimal TG from the Bloch‐Siegert acquisition (bottom), showing good correlation.
