# Supplementary material for: Evaluation of an integrated variable flip angle protocol to estimate coil B1 for hyperpolarized MRI
Source: Magn Reson Med. 2024 Nov 17;93(4):1615–28. doi: 10.1002/mrm.30378 (PMC11782732; doi:10.1002/mrm.30378)
Supplement: Supplementary file 1 — Figure S1: T1 estimation errors using the CFA approach with fifteen 10° acquisitions (top) and VFA approach with five 10°, five 20°, and five 30° acquisitions (bottom). T1 was varied with the TR kept at 0.23 s (left), and TR was varied with the T1 kept at 23 s. Figure S2: Signal train from a hyperpolarized carbon‐13 phantom using a short TR (left) and a long TR (right), showing that T1 agrees with literature value 51 if TR and total acquisition time is long enough. Figure S3: B1 maps from five hyperpolarized carbon‐13 VFA scans, showing mean ΔB1 values close to 1. Figure S4: B1 maps from the hyperpolarized Xenon‐129 VFA scans (top, middle rows), scatter plot of mean ΔB1 against the TG used divided by the optimal TG from the Bloch‐Siegert acquisition (bottom), showing good correlation. [file MRM-93-1615-s001.docx]

# **Supplementary Material**

## **Figure S1T1 Estimation**

Simultions and spectroscopy of a hyperpolarized carbon-13 phantom (hyperpolarized [1-^13^C]pyruvate in a falcon tube) show that simultaneous T_1_ and B_1_ measurement with the VFA method can be acheived with long enough TR relative to T_1_.


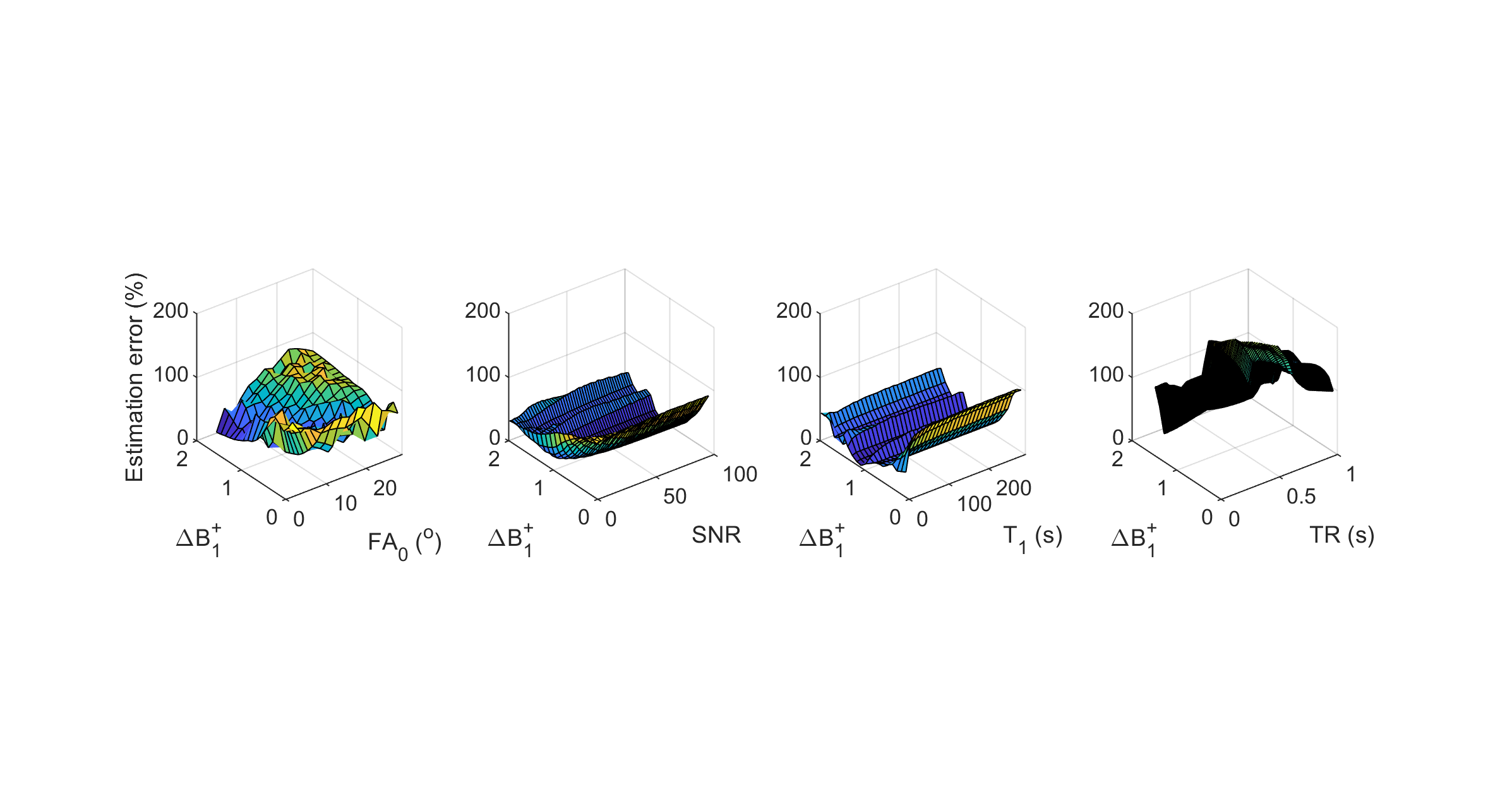

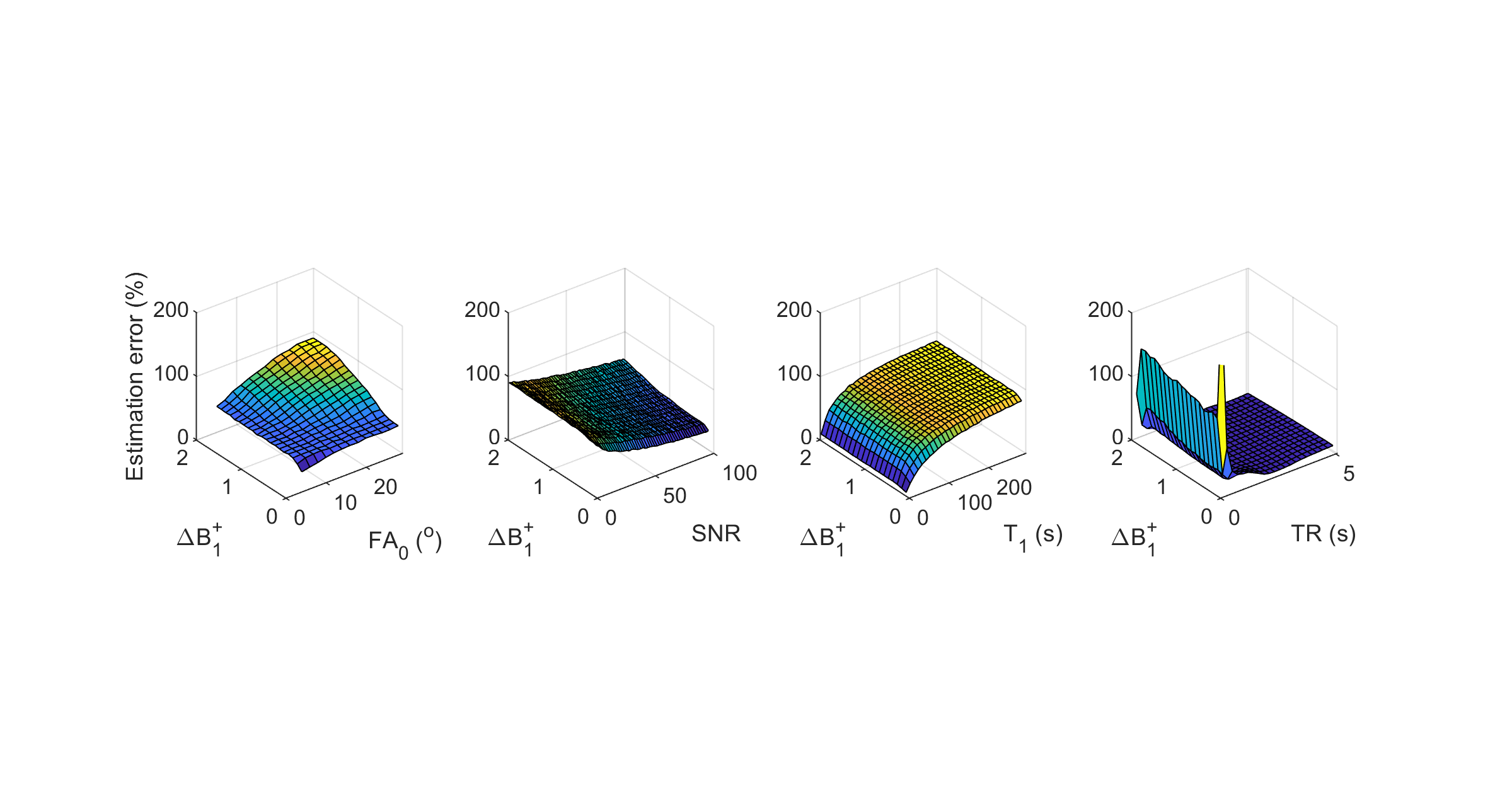


Figure S1 T1 estimation errors using the CFA approach with fifteen 10^o^ acquisitions (top) and VFA approach with five 10^o^, five 20^o^, and five 30^o^ acquisitions (bottom). T_1_ was varied with the TR kept at 0.23s (left), and TR was varied with the T_1_ kept at 23s.

**Figure S2**


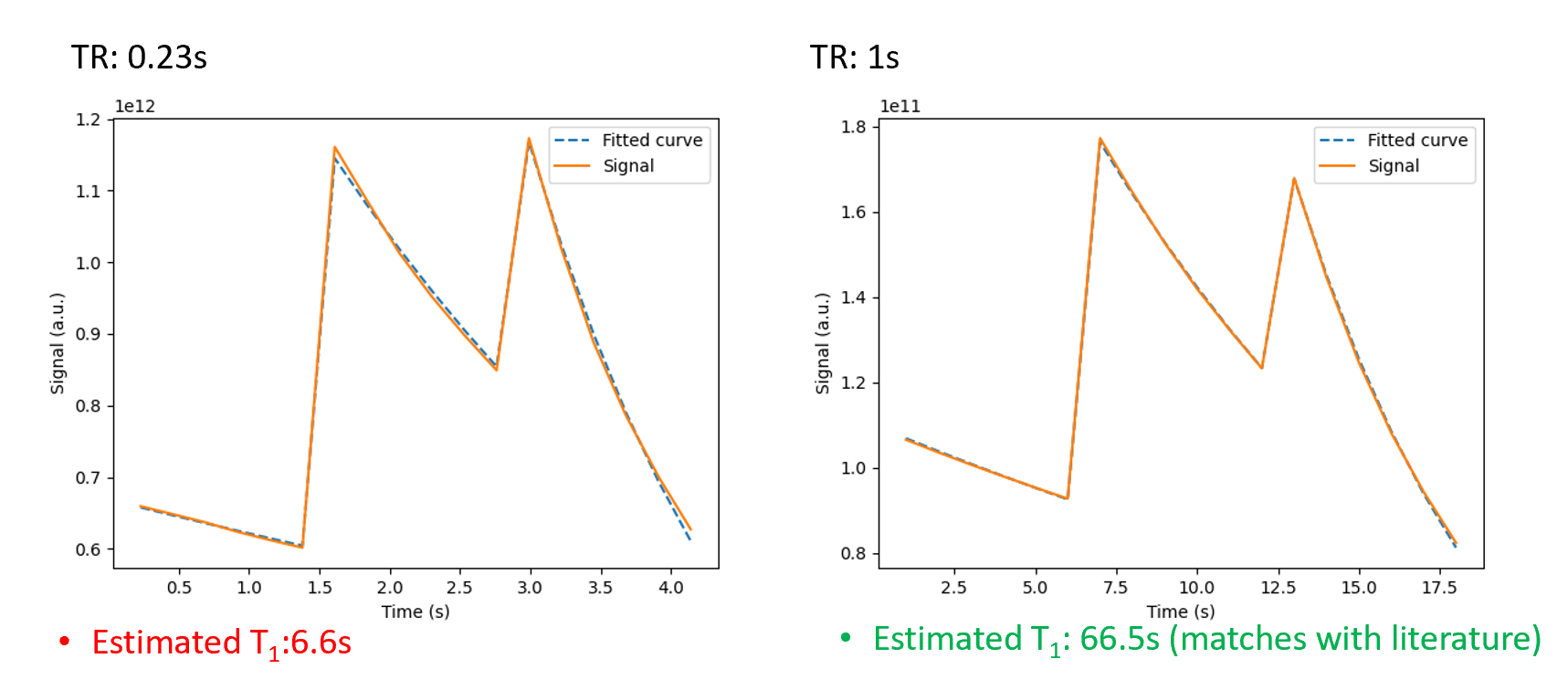


Figure S2 Signal train from a hyperpolarized carbon-13 phantom using a short TR (left) and a long TR (right), showing that T_1_ agrees with literature value^51^ if TR and total acquisition time is long enough.

In a spectroscopic acquisition of a carbon-13 phantom, the T_1_ could not be estimated with a 4-second acquisition, but a T_1_ close to the literature value could be estimated when the acquisition was extended to 18 seconds.

However, this acquisition time is not achievable in vivo since the T_1_ is quite long relative to the available imaging time. Moreover, the actual in vivo acquisition is confounded by many factors, especially in the hyperpolarized carbon-13 case where there is inflow and outflow of hyperpolarized bolus, as well as the continuous conversion of the metabolites.

## **Figure S3 Comparison with Bloch-Siegert**

Figure S3 B_1_ maps from five hyperpolarized carbon-13 VFA scans, showing mean ΔB_1_ values close to 1.

In the hyperpolarized C-13 experiments, the B_1_ maps were acquired using the TG that was determined from a prior measurement using the urea phantom. Therefore, the average B1 value obtained were quite close to 1. Note that the calibration using the urea phantom should be treated as a rough estimation only, due to its heavy dependence on the position of the urea phantom relative to the coil and the field of view.

**Figure S4**


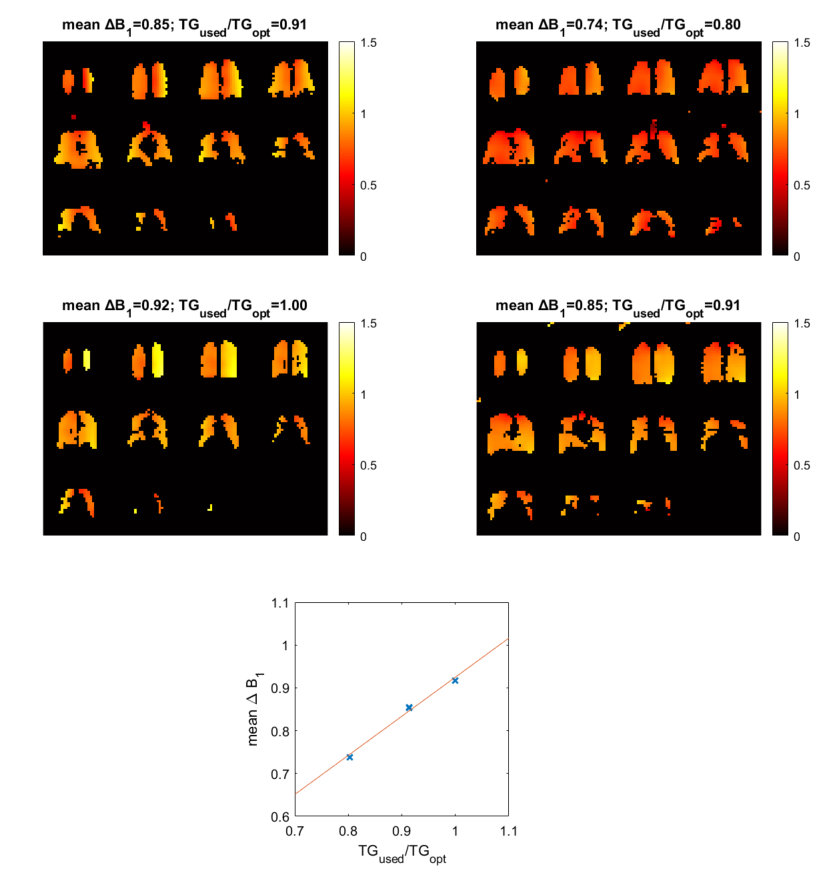


Figure S4 B_1_ maps from the hyperpolarized Xenon-129 VFA scans (top, middle rows), scatter plot of mean ΔB_1_ against the TG used divided by the optimal TG from the Bloch-Siegert acquisition (bottom), showing good correlation.

For the hyperpolarized Xenon-129 experiments, the TG was not adjusted per participant because the B_1_ mapping was performed within the same calibration scan. When a lower ΔB_1_ value was measured, a higher TG is requested to hit a 90^o^ flip angle. There is a strong correlation (R^2^ =0.99).
